# Interval cancer after two rounds of a Swedish population-based screening program using gender-specific cut-off levels in fecal immunochemical test

**DOI:** 10.1177/09691413231185722

**Published:** 2023-07-16

**Authors:** Hanna Ribbing Wilén, Johannes Blom

**Affiliations:** 1 Department of Clinical Science and Education Södersjukhuset (KI SÖS), 27106 Karolinska Institutet, Stockholm, Sweden

**Keywords:** Colorectal cancer, screening, fecal immunochemical test, FIT, interval cancer, gender

## Abstract

**Objective:**

To evaluate interval cancer (IC) after two screening rounds of the Swedish population-based screening program of Stockholm–Gotland applying gender-specific cut-off levels in the fecal immunochemical test (FIT).

**Methods:**

All 60- to 69-year-olds invited to screening 2015–2019 were included. The cut-off level for a positive test was 40 µg/g in women and 80 µg/g in men. Screening-detected colorectal cancers (SD CRCs) and ICs were verified in the Swedish Colorectal Cancer Register, and the follow-up time was two years from invitation. The test sensitivity, the IC rate (ICs per 10,000 screening negatives) and the IC incidence (ICs per 100,000 person-years) relative to the background CRC incidence were assessed by gender and age. The FIT levels were compared in men and women for CRCs diagnosed within one year of the sample.

**Results:**

In the second screening round, 229,187 were invited, and SD CRCs and ICs were diagnosed in 193 and 144, respectively. The IC rate was 8.9 (7.4–10.3) and test sensitivity 0.61 (0.55–0.66), and was similar in men and women. For two screening rounds, the IC rate was significantly higher in men than in women, but the IC incidence/ background CRC incidence was similar in both genders. The FIT levels in female participants with CRC were significantly lower overall, and in early-staged CRCs as compared to men, and proximal localization was more common in women. In multivariable analysis, FIT levels were significantly lower in proximal CRCs.

**Conclusion:**

Over two rounds, the IC incidence relative to the background CRC incidence was similar in men and women supporting a gender-specific screening strategy. The results could be explained by lower FIT levels in women due to proximal CRC localization.

## Introduction

The purpose of colorectal cancer (CRC) screening is to reduce the disease-specific mortality. Screening with the guaiac fecal occult blood test (gFOBT) has demonstrated an overall 16% decrease in mortality in randomized controlled trials.^1^ In recent years, many programs have switched from gFOBT to fecal immunochemical test (FIT) with a higher sensitivity for advanced neoplasia.^2,3^

Interval cancer (IC), that is, CRCs diagnosed after a negative screening episode and before invitation to the next screening round, and the test sensitivity are important quality measures of a screening program that will affect the ability of the program to reduce CRC mortality. Since the rate of IC is dependent on the background CRC incidence and the screening interval, the European guidelines recommend that the IC rate is reported in person-years of follow-up and as a proportion of the background incidence.^4,5^

Several ongoing FIT screening programs have reported a lower test sensitivity and a higher proportional IC rate in women than in men.^6–9^ The CRC prevalence is higher in men and the FIT positivity is lower in women, contributing to the gender differences in sensitivity and IC rate seen in FIT screening. The reason for the lower FIT in women is unclear but could be related to a higher rate of lesions in the proximal colon that are less prone to bleeding or a longer colonic transit time in women, leading to a larger degree of fecal hemoglobin degradation.^10–13^ If the lower FIT in women was due to obstipation, the gender difference would be greater in CRCs in the proximal colon than in distal segments. On the contrary, a study from the Scottish bowel screening program showed significantly lower FIT levels in women only in the left-sided CRCs. However, the Scottish study was only conducted in screening-detected CRCs with FIT ≥80 µg/g.^14^

Gender-specific FIT screening has been proposed to achieve equal positivity in men and women but is currently only used in Finland and Sweden.^15–17^ We recently reported the first screening round in the Swedish population-based program of Stockholm–Gotland applying cut-off levels of 40 µg/g in women and 80 µg/g in men, and demonstrated a higher test sensitivity in women than in men and a tendency towards a higher IC rate in men when compared to the background CRC incidence.^18^

The aim of this study was to assess the IC incidence and test sensitivity in men and women after two screening rounds to achieve a higher precision in the estimates. A secondary aim was to investigate FIT levels in men and women diagnosed with CRC including participants with FIT levels below 80 µg/g.

## Methods

### Study population

This study was undertaken in the Swedish region of Stockholm–Gotland, where organized population-based CRC screening with biennial gFOBT was implemented in 2008 and fully expanded in 2012.^19^ Since 2015, the program applies a gender-specific FIT screening strategy with a cut-off level for a positive test of 40 µg/g in women and 80 µg/g in men.^17^ The program invited all 60- to 69-year-olds residing in the region without exclusions, except participants from previous rounds referred to an adenoma surveillance program or surgery. The present study cohort comprises all invited to the second screening round of the FIT program, from October 2017 to September 2019, and a pooled analysis with those invited to the first screening round, from October 2015 to September 2017.^18^ The follow-up time was two years from screening invitation, that is, until the next screening round.

### FIT

All invitations were managed by the Regional Cancer Centre in Stockholm and included a FIT test kit (OC sensor Eiken, Japan) along with the invitation. The participants were instructed to document the sample date on the test tube and send it to the laboratory as soon as possible in a prepaid envelope. A new kit was sent in the case of an unanalyzable result, and a reminder was sent after 8 weeks in the case of no response. Women with FIT ≥40 µg/g and men with FIT ≥80 µg/g were considered FIT positive and offered a diagnostic follow-up colonoscopy. Failure to provide a complete test result during the study period was considered as non-participation. The invitation, sample date, date of analysis, and test results were recorded in the screening register. If more than one test result was documented in the register during the screening round of 2 years due to, for example, an early invitation for the third round, the first dated sample was included in the analysis.

### Colonoscopies

All screening colonoscopies until March 2019 were recorded in the screening register, but from April 2019, all screening colonoscopies were registered in the Swedish quality register for colonoscopies and colorectal cancer screening (SveReKKS). The databases provided information on colonoscopy quality parameters, colonoscopy findings and date of the examinations. Colonoscopy compliance was defined as having had a colonoscopy after a positive FIT within the study period.

### Colorectal cancers

All CRCs among the invitees were verified in the Swedish Colorectal Cancer Register (SCRCR), which has a coverage of 99% and an overall validity of 90%.^20^

A screening-detected CRC (SD CRC) was defined as a CRC diagnosed after a positive FIT and positive screening colonoscopy or within 6 months of the screening examination, that is, the CRC was detected at an early follow-up colonoscopy initiated because of the screening colonoscopy. An interval CRC (IC) was defined as a CRC diagnosed after a negative FIT (FIT IC) or a positive FIT and a negative colonoscopy (colonoscopy IC), or after a positive FIT in those not compliant with colonoscopy (IC non-compliant to colonoscopy), and before the next screening invitation (2 years). CRC diagnosed in non-participants was not considered as IC.^5^

The histopathological TNM stage recorded in the SCRCR was used to determine the CRC stage, but in the case of missing data, the preoperative clinical TNM was assessed. CRC stage was defined according to the TNM classification of malignant tumors (8^th^ edn, 2017) and further grouped into stages I–II and III–IV, respectively.^21^

### Statistics

Age was defined as age at screening invitation rounded to whole years and grouped into 60–64 and 65–69, respectively. The positive predictive value (PPV) was defined as the number of SD CRC divided by the number of FIT positives in each subgroup. Confidence intervals (CI) were calculated for the proportion assuming independent sample and a normal distribution. The test sensitivity was defined as SD CRC divided by the sum of SD CRC and CRC diagnosed within 2 years of a negative FIT (FIT IC). Hence, the term test sensitivity refers to the mode of CRC detection among the participants identified in the SCRCR, disregarding that a proportion of the ICs could have been fast-growing de novo cancers not present at the time of FIT sampling.

IC rate was defined as the number of FIT ICs, colonoscopy ICs and ICs in non-compliers to colonoscopy per 10,000 FIT negatives or FIT positives without SD CRC, that is, the screening negatives. The IC incidence rate was calculated as the number of total ICs per 100,000 person-years of follow-up with regards to CRC diagnosis among the screening negatives, that is, 2 years per individual unless an IC was diagnosed within 2 year of screening invitation. The person-years and the ICs from rounds one and two were summarized. The experienced incidence rate (EIR) was calculated as the mean incidence of CRC per 100,000 in each subgroup in the 10 years preceding CRC screening initiation in the Stockholm–Gotland region, that is, 1998–2007. The 95% CI for the rate ratio was calculated using the MedCalc online calculator.^22^ All other analyses were done in R version 4.1.0.^23^

The number of FIT ICs, the IC rate, and the test sensitivity were estimated for a cut-off level of 80 µg/g in both genders, assuming SD CRCs, colonoscopy ICs, and CRCs in those non-compliant to colonoscopy in women with FIT 40–79 µg/g would all have been classified as FIT ICs.

The FIT levels and CRC characteristics were evaluated in men and women with SD CRC or IC diagnosed within 1 year of the FIT sample. A multivariable quantile regression was performed on the median FIT level including the variables sex, stage, pT stage, and localization. A separate univariate sensitivity analysis was performed in all men and women with SD CRC and FIT level ≥80 µg/g.

Differences in PPV, test sensitivity, and IC rate in subgroups were assessed using the chi-squared test. This was also used to examine stage and gender variation in the separate groups of CRCs among participants and non-participants. Differences in FIT levels between men and women and different CRC characteristics are presented as median and interquartile range and were assessed with the Wilcoxon rank sum test. A *p* <0.05 was considered statistically significant.

The study was approved by the Ethics Review Authority (no. 2019-04850, 2023-00400-02). Information about the screening register and how to unregister was included in the invitation and reply letter. Informed consent was asked for at the endoscopy unit for the registration in SveReKKS. Access to underlying research material can be obtained by email to the corresponding author.

## Results

In the second FIT screening round from October 2017 to September 2019, 229,187 individuals were invited, of whom 164,528 (59.0%) had been invited to the previous FIT screening round. Overall, 70.9% participated, and the FIT participation rate was higher in women than in men. The FIT positivity rate was 2.3%, and overall compliance to colonoscopy was 86.7%. In the second screening round, 193 SD CRCs were detected (82 in women and 111 in men), which rendered a PPV of 5.1%. The PPV was significantly higher in men than in women. Participation, FIT positivity and PPV in different age and gender subgroups for the first and second screening rounds are detailed in Table 1. The total follow-up time in the second round was 324 507 person-years in the screening negatives.

**Table 1.** Participation and positive predictive values in men and women invited to screening in the Stockholm–Gotland screening program in the first two screening rounds 2015–2017 and 2017–2019.

Age at invitation and gender	FIT participants 2015–2017 *n* (%)	FIT positive 2015–2017 *n* (%)	SD CRC 2015–2017 *n*	PPV 2015–2017 % (95% CI)*	FIT participants 2017–2019 *n* (%)	FIT positive 2017–2019 *n* (%)	SD CRC 2017–2019 *n*	PPV 2017–2019 % (95% CI)**
Women <65	46 460 (70.3)	1201 (2.6)	59	4.9 (3.7–6.1)	52 740 (73.1)	1148 (2.2)	48	4.2 (3.0–5.3)
Women ≥65	31 680 (74.0)	1011 (3.2)	63	6.2 (4.7–7.7)	32 412 (74.5)	809 (2.5)	34	4.2 (2.8–5.6)
Men <65	42 159 (63.8)	1046 (2.5)	72	6.9 (5.3–8.4)	48 816 (67.5)	1105 (2.3)	67	6.1 (4.7–7.5)
Men ≥65	26 679 (67.7)	799 (3.0)	63	7.9 (6.0–9.8)	28 504 (69.1)	694 (2.4)	44	6.3 (4.5–8.2)
All	146 978 (68.6)	4057 (2.8)	257	6.3 (5.6–7.1)	162 472 (70.9)	3756 (2.3)	193	5.1 (4.4–5.8)

FIT: fecal immunochemical test; CRC: colorectal cancer; FIT participants %: number of individuals with a valid FIT divided by number of individuals invited; FIT positive: FIT ≥40 µg/g in women and 80 µg/g in men; SD CRC: screening-detected CRC; PPV: positive predictive value; SD CRC/FIT positives; CI: confidence interval.

* *p* = 0.023 for difference in PPV between all men and women. *p*>0.05 for difference in PPV between all <65 and all ≥65, between men and women <65, and between men and women ≥65.

** *p* = 0.008 for difference in PPV between all men and women. *p*>0.05 for difference in PPV between men and women <65 years, between men and women ≥65 years, and for difference in PPV between all <65 and all ≥65 years.

Due to slightly less than 24 months between screening invitations for the first and second FIT rounds, 10 of the individuals with FIT IC in the first round were reclassified to FIT negatives (*n* = 10) in round one, and classified as SD CRC (*n* = 8), IC non-compliant to colonoscopy (*n* = 1), and non-participant (*n* = 1) in round two.

In the second screening round, there were 144 participants with ICs diagnosed within 2 years of invitation: 125 FIT ICs, 3 colonoscopy ICs, and 16 in those non-compliant to colonoscopy. In Supplemental Table 1, the number of invited, the FIT negatives, the number of colonoscopies and the ICs are detailed for different age and gender groups in the second screening round.

 Table 2 displays the FIT ICs, the IC rate, and the test sensitivity in age and gender subgroups for the first and second round, and for the two rounds summarized. In the second round, the IC rate in all women was 7.5 (5.7–9.4) and that in men 10.4 (8.1–12.6), *p* = 0.067. The test sensitivity was similar in women and men in the second round: 0.58 (0.50–0.66) as compared to 0.63 (0.56–0.70), *p* = 0.477.

**Table 2.** FIT IC, IC rate and test sensitivity in different age and gender subgroups in the Stockholm–Gotland screening program for two screening rounds 2015–2017 and 2017–2019.

Age at invitation and gender	FIT IC 2015–2017	IC rate 2015–2017 (95% CI)	Test sensitivity 2015–2017 (95% CI)	FIT IC 2017–2019	IC rate 2017–2019 (95% CI)*	Test sensitivity 2017–2019 (95% CI)**	FIT IC 2015–2019	IC rate 2015–2019 (95% CI)***	Test sensitivity 2015–2019 (95% CI)****
Women <65	19	4.5 (2.6–6.5)	0.76 (0.66–0.85)	27	5.9 (3.8–8.0)	0.64 (0.53–0.75)	46	5.2 (3.8–6.7)	0.70 (0.63–0.77)
Women ≥65	20	7.6 (4.6–10.6)	0.76 (0.67–0.85)	32	10.2 (6.7–13.7)	0.52 (0.39–0.64)	52	8.9 (6.6–11.2)	0.65 (0.57–0.73)
Men <65	32	8.6 (5.8–11.3)	0.69 (0.60–0.78)	35	9.0 (6.4–11.7)	0.66 (0.56–0.75)	67	8.8 (6.9–10.7)	0.67 (0.61–0.74)
Men ≥65	43	16.2 (12.3–20.0)	0.59 (0.50–0.69)	31	12.6 (8.5–16.8)	0.59 (0.48–0.70)	74	14.3 (11.2–17.5)	0.59 (0.52–0.66)
All	114	8.5 (7.0–9.9)	0.69 (0.65–0.74)	125	8.9 (7.4–10.3)	0.61 (0.55–0.66)	239	8.7 (7.6–9.7)	0.65 (0.62–0.69)

FIT: fecal immunochemical test; IC: interval cancer; FIT IC: IC after negative FIT; colonoscopy IC: IC after negative screening colonoscopy; IC rate: number of IC (FIT IC, colonoscopy IC or IC in those non-compliant to colonoscopy) per 10,000 FIT negatives or FIT positives with negative or no colonoscopy; SD CRC: screening-detected CRC; test sensitivity: SD CRC/(SD CRC + FIT IC).

* *p* = 0.012 for difference in IC rate between all <65 years and ≥65. *p* = 0.036 for difference in IC rate between women <65 and women ≥65 years.

** *p* > 0.05 for difference in test sensitivity between men and women and between <65 years and ≥65 years.

*** *p* = 0.000092 for difference in IC rate between all men and women. *p* = 0.000052 for difference in IC rate between all <65 and ≥65 years. *p* = 0.007 for difference in IC rate between women <65 and women ≥65 years. *P* = 0.002 for difference in IC rate between men <65 and men ≥65 years. *p* = 0.004 for difference in IC rate between men and women <65 years. *p* = 0.007 for difference in IC rate between men and women ≥65 years.

**** *p* > 0.05 for difference in test sensitivity between all men and women and between participants <65 and ≥65 years of age.

Over two screening rounds, the IC rate in women was 6.7 (5.4–7.9) as compared to 10.9 (9.2–12.6) in men, *p* = 0.000092. The IC rate was significantly higher in men and women ≥65 years than in participants <65 years of age. The test sensitivity in women was 0.68 (0.62–0.73) and in men 0.64 (0.59–0.68), *p* = 0.313. There was no difference in test sensitivity between the older and the younger age groups.

In Supplemental Table 2, the FIT participants, the FIT positives and negatives, the number of colonoscopies, the SD CRCs, and the ICs from screening rounds one and two are summarized.

Estimations of FIT IC, IC rate and test sensitivity by different age and gender subgroups with a cut-off level of 80 µg/g for both genders are displayed in Supplemental Table 3 for the first, the second, and both screening rounds. A cut-off level of 80 µg/g for both men and women would have rendered similar IC rates in men and women in both screening rounds but a significantly lower test sensitivity in women in the second round and in the two rounds summarized.

 Table 3 displays the IC incidence rate in relation to the EIR in different subgroups for the second screening round, and summarized for rounds one and two. For the two screening rounds, the IC incidence rate was 26–72 per 100 000 person-years and increased with age and male gender. When compared to the EIR, the IC incidence was 0.34–0.36 and similar in men and women and for all age categories.

**Table 3.** IC incidence rate in relation to experienced incidence rate (EIR) in the Stockholm–Gotland screening program 2017–2019 and 2015–2019.

Age at invitation and gender	Total ICs 2017–2019, N	Person-years of follow-up, 2017–2019	IC incidence rate 2017–2019 (95% CI)	EIR	IC incidence rate/EIR 2017–2019 (95% CI)	Total ICs 2015–2019, N	Person-years of follow-up, 2015–2019	IC incidence rate 2015–2019 (95% CI)	IC incidence rate/EIR 2015–2019 (95% CI)
Women <65	31	105 376	29.4 (20.0–41.8)	78	0.38 (0.24–0.58)	52	198 171	26.2 (19.6–34.4)	0.34 (0.23–0.48)
Women ≥65	33	64 746	51.0 (35.1–71.6)	132	0.39 (0.26–0.57)	57	127 974	44.5 (33.7–57.7)	0.34 (0.24–0.46)
Men <65	44	97 477	45.1 (32.8–60.6)	124	0.36 (0.25–0.52)	80	181 640	44.0 (34.9–54.8)	0.36 (0.26–0.47)
Men ≥65	36	56 908	63.3 (44.3–87.6)	198	0.32 (0.22–0.46)	79	110 125	71.7 (56.8–89.4)	0.36 (0.28–0.47)

CRC: colorectal cancer; IC: interval CRC; Person-years of follow-up for screening negatives regarding CRC diagnosis; IC incidence rate: number of ICs among FIT negatives or FIT positives with negative or no colonoscopy per 100 000 person-years of follow-up; EIR: experienced incidence rate; mean CRC incidence in Stockholm–Gotland region for the years 1998–2007 in different age and gender groups per 100 000. CI: confidence interval.

Among those invited to screening between 2015 and 2019, there were 1040 individuals diagnosed with CRC: 428 women and 612 men. Of all the invitees with CRCs, 322 were non-participants. Stages I and II CRC were significantly more common in participants than in non-participants; 366 (57%) vs 107 (40%). There was a significantly higher proportion of proximally located CRC in women than in men; 186 (43%) and 175 (29%), respectively (Supplemental Table 4).

The FIT levels and CRC characteristics in men and women with SD CRC or ICs diagnosed within 1 year of the FIT sample (*n* = 552) are listed in Table 4. Overall, the median FIT level was lower in women than in men (*p* = 0.011). Women with CRC stages I and II or CRCs T1 and T2 had significantly lower FIT levels than men, but FIT levels were similar in men and women with higher staged CRCs. There was a tendency towards lower FIT levels in women with proximal CRC than in men (*p* = 0.054). In all men and women, the median FIT level in proximal and distal CRC was 115 and 271.5 µg/g, respectively (*p* = 0.0000041). A multivariable quantile regression was performed on the median FIT level including the variables sex, stage, pT stage, and localization. Proximally located CRC remained independently associated with lower median FIT levels (*p* = 0.0051) and pT3 (but not pT4) with a higher median FIT level (*p* = 0.0394). No significant gender differences were seen in FIT levels for CRC localization or stage in a univariate sensitivity analysis of all men and women with SD CRC and FIT level ≥80 µg/g (*n* = 402) (Supplemental Table 5).

**Table 4.** FIT levels in men and women diagnosed with CRC within 1 year of FIT screening sample.

CRC characteristics	Women, *N* (% of women)	Men, *N* (% of men)	Women, FIT median (IQ range)	Men, FIT median (IQ range)	*p*-value*
Total	239	313	153 (54.5–798.5)	265 (104–849)	0.011
					
Stages I&II	128 (54)	173 (55)	146.5 (58.8–563.8)	262 (120–793)	0.005
Stages III&IV	92 (38)	111 (35)	219.5 (52.5–1037)	308 (84–1232)	0.489
No stage	19 (7.9)	29 (9.3)	-	-	-
					
pT1	44 (18)	71 (23)	107 (55.5–329)	169 (115–435)	0.032
pT2	52 (22)	52 (17)	117 (52.8–393.3)	417.5 (133.8–895.5)	0.001
pT3	78 (33)	102 (33)	421 (89.3–1599.8)	349.5 (118.8–1476.2)	0.892
pT4	31 (13)	24 (7.7)	190 (18.5–932)	217.5 (25.8–743.8)	0.812
No pT stage, TX or T0	34 (14)	64 (20)	-	-	-
					
Proximal	88 (37)	79 (25)	89.5 (40.8–370.5)	159 (44–823.5)	0.054
Distal	149 (62)	233 (74)	221 (71–1003)	294 (124–851)	0.341
No localization	2 (0.8)	1 (0.3)	-	-	-

FIT: fecal immunochemical test; CRC: colorectal cancer; Stages I&II: no regional lymph node metastasis; stages III&IV: regional lymph node or distant metastases; pT: histopathological T stage; Proximal: caecum to splenic flexure; Distal: descending colon to rectum; IQ range: interquartile range.

* Wilcoxon rank sum test of FIT level in men versus women.

## Discussion

This study was the first evaluation of multiple rounds in a population-based FIT screening program that applies a gender-specific screening strategy, and demonstrated a similar test sensitivity and IC incidence compared to the background incidence in men and women. Furthermore, the FIT levels in participating women with CRC were significantly lower, especially in the early-staged CRCs, as compared to participating men with CRC, which may explain the less beneficial results in women seen in gender-uniform FIT screening.

The IC rate was similar in men and women in the second screening round, as opposed to the first screening round in which the IC rate was significantly higher in men. As the gFOBT program preceded FIT screening in the region of Stockholm–Gotland, the screening population was not screening naïve in the first FIT screening round. The switch from gFOBT to FIT screening lowered the IC rate more in women than in men, and from first to second FIT round more in men than in women. This might be explained by the preceding gFOBT screening being less effective in women, leading to a higher level of detection of CRCs in women after switching to FIT and, hence, a lower IC rate. By the second screening round, this effect in women was reduced, and the decline in IC rate was mainly seen in the older age group of men. Taking both rounds into account, the IC rate was still significantly higher in men than in women.

The IC rate is determined both by the screening strategy and by the background CRC incidence, and therefore, it is important to relate the IC rate to the background incidence.^5^ The CRC incidence is lower in women as compared to men, which leads to a lower number of ICs in women. Over multiple screening rounds and at different cut-off levels of FIT, several studies have shown a consistently lower positivity rate in women than in men, hence a higher rate of FIT-negative women.^24–26^ Since the IC rate is measured as the number of ICs among the test negatives, the result would be a lower IC rate in women.^27^ In the present study, we estimated the IC rate with a cut-off level of 80 µg/g in both genders and found similar IC rates in men and women, indicating this screening strategy to be less beneficial in women. With the gender-specific screening strategy in the Stockholm–Gotland screening program and when taking both rounds and the background incidence (EIR) into account, the IC incidence was similar in men and women, and the program detected approximately 65% of the incident CRCs before screening implementation in the region in all age and gender categories.

The test sensitivity, referring to the proportion of SD CRC of the total number of SD CRC and FIT IC, was around 61% in the second screening round, and comparable between men and women both in the second screening round and over two screening rounds. The higher test sensitivity in women in the first round was possibly due to the higher detection of CRC in women as compared to men when switching from gFOBT to FIT as previously described. The test sensitivity is expected to decline with multiple rounds as most CRC are detected in the first round leading to a selection of low-risk individuals in subsequent rounds.^24,26,28^ However, as gFOBT only has a sensitivity for advanced adenoma of around 10% (lower in women than in men), the first FIT round could be viewed as a prevalent screening round for precursors to CRC.^29,30^ Moreover, the gFOBT program in Stockholm–Gotland demonstrated a significantly lower test sensitivity for CRC in women than in men; so, the gain in sensitivity switching from gFOBT to FIT was more pronounced in women (+43–53%) than in men (+6–23%).^18,31^ The second FIT round could therefore be considered as an incident round and explain the lower detection of CRC, particularly in women, as compared to the first.

When evaluating participants diagnosed with CRC within 1 year of FIT participation, we found a lower median FIT level in women as compared to men. This could be explained by the higher rate of proximal CRCs seen in women as the FIT levels were lower in proximal than in distal CRCs. Moreover, the FIT levels were particularly low in women with early-stage CRC (stages I and II, T1 and T2) as compared to the FIT levels in men. The gender differences disappeared in more advanced staged CRCs, as they did when restricting the analysis to only those with CRC and FIT levels above 80 µg/g. Previous studies have found an association between T stage and FIT levels because the tumor depth is more determinant for the fecal blood loss in the bowel lumen than the existence of regional or distant metastases.^32^ Early CRCs are weaker sources of bleeding, in which the lesser amount of colonic blood loss in women as compared to men becomes apparent. This is an important finding as the aim of screening is to diagnose CRC at an early stage to be able to improve treatment and, eventually, mortality, which seems to warrant lower cut-off levels for women.

Neither our findings nor the results from the Scottish bowel screening program seem to support the theory that the lower fecal blood levels in women are explained by a longer colonic transit time and thereby an excess degradation of hemoglobin as compared to men, since FIT levels in participants with CRC in the proximal segments of the colon were similar in men and women.^14^ However, the FIT levels varied considerably, and in a larger cohort, gender differences in FIT levels of distinct colonic localizations might become evident.

This study has several limitations. Firstly, the analysis of FIT levels and CRC characteristics in men and women included a time span from FIT sample to CRC diagnosis of up to 1 year. Probably, although difficult to monitor, the FIT levels in those with CRC progress as the CRC progresses with time. Ultimately, the FIT sample should be collected just before the CRC diagnosis. However, in order to assess the lower FIT levels in both men and women, it was necessary to include the FIT ICs in the analysis. Since the cut-off level is lower in women, the CRCs with low FIT levels were more often SD CRCs in women and FIT ICs in men, and the SD CRCs were diagnosed more recently after the FIT sample was taken, which could lead to an underestimation of FIT levels in the FIT ICs. As FIT ICs are more often proximally located compared to SD CRCs, this could have underestimated the differences in men and women.^8,18,33^

Secondly, misclassification of FIT ICs and SD CRCs could have occurred in the second round since this was discovered in the first round. A few with FIT IC in the first round were in fact invited again slightly earlier than 24 months after previous invitation, and thus reclassified to SD CRC in the second round. However, this problem was limited and non-differential between men and women.

Thirdly, the EIR was based on incidence numbers before gFOBT screening was initiated in the region in 2008 and does not account for time trends in CRC incidence that might have occurred after that. However, statistics from the National Board of Health and Welfare show that the CRC incidence in 60- to 69-year-olds has been fairly stable in Sweden between 2009 and 2019.^34^ Moreover, the EIR was the mean CRC incidence per 100,000 in different age and gender groups, which is slightly different from the screening cohort IC incidence in 100,000 person-years, because during follow-up, this cohort gets older and CRC incidence increases with age. This could have led to an overestimation of the rate ratio.

The strength of this study was the evaluation of a population-based screening program with 100% coverage and all CRCs verified in the SCRCR. With data now available for two rounds, this enabled greater precision of the estimates. The results are to a large extent generalizable to other regions, which is important since the gender-specific screening strategy is now implemented throughout Sweden.

In conclusion, this study over two screening rounds from the Swedish region of Stockholm–Gotland, with cut-off levels of 40 µg/g in women and 80 µg/g in men, demonstrated that the test sensitivity and the IC incidence relative to the background CRC incidence were similar in men and women. Our results confirm the conclusions from the first screening round and support the continued use of a gender-specific screening strategy, but further evaluations by screening rounds are required.

## Supplemental Material

sj-docx-1-msc-10.1177_09691413231185722 - Supplemental material for Interval cancer after two rounds of a Swedish population-based screening program using gender-specific cut-off levels in fecal immunochemical test Supplemental material, sj-docx-1-msc-10.1177_09691413231185722 for Interval cancer after two rounds of a Swedish population-based screening program using gender-specific cut-off levels in fecal immunochemical test by Hanna Ribbing Wilén and Johannes Blom in Journal of Medical Screening
