# Supplementary material for: Interval cancer after two rounds of a Swedish population-based screening program using gender-specific cut-off levels in fecal immunochemical test
Source: J Med Screen. 2023 Jul 16;31(1):8–14. doi: 10.1177/09691413231185722 (PMC10878001; doi:10.1177/09691413231185722)
Supplement: sj-docx-1-msc-10.1177_09691413231185722 - Supplemental material for Interval cancer after two rounds of a Swedish population-based screening program using gender-specific cut-off levels in fecal immunochemical test [file sj-docx-1-msc-10.1177_09691413231185722.docx]

Supplementary Table 1. Number of invited, FIT results, SD CRC and ICs in different age and gender subgroups in the Stockholm-Gotland screening program 2017-2019.

| Age at invitation and gender | Invited,  N | FIT Participants | FIT negatives | FIT positives | Colono-scopies | SD CRC | FIT IC | Colonoscopy IC | IC non-compliant to colonoscopy |
| --- | --- | --- | --- | --- | --- | --- | --- | --- | --- |
| Women <65 | 72 135 | 52 740 | 51 592 | 1148 | 1011 | 48 | 27 | 0 | 4 |
| Women ≥65 | 43 502 | 32 412 | 31 603 | 809 | 708 | 34 | 32 | 1 | 0 |
| Men <65 | 72 290 | 48 816 | 47 711 | 1105 | 932 | 67 | 35 | 1 | 8 |
| Men ≥65 | 41 260 | 28 504 | 27 810 | 694 | 604 | 44 | 31 | 1 | 4 |
| All | 229 187 | 162 472 | 158 716 | 3756 | 3255 | 193 | 125 | 3 | 16 |

FIT= Fecal Immunochemical Test. SD CRC= screening detected CRC. IC= interval cancer. FIT IC= IC after negative FIT. Colonoscopy IC= IC after negative screening colonoscopy. IC rate= number of IC per 10 000 FIT negatives or FIT positives with negative or no colonoscopy. Test sensitivity = SD CRC/(SD CRC+FIT IC).

Supplementary Table 2. Participants, FIT results, SD-CRC and ICs in different age and gender subgroups in the Stockholm-Gotland screening program 2015-2019.

| Age at invitation and gender | FIT Participants  2015-2019 | FIT negatives  2015-2019 | FIT  positives 2015-2019 | Colono-scopies  2015-2019 | SD-CRC 2015-2019 | FIT IC  2015-2019 | Colonoscopy IC  2015-2019 | IC non-compliant to colonoscopy  2015-2019 |
| --- | --- | --- | --- | --- | --- | --- | --- | --- |
| Women <65 | 99 200 | 96 851 | 2349 | 2054 | 107 | 46 | 2 | 4 |
| Women ≥65 | 64 092 | 62 272 | 1820 | 1609 | 97 | 52 | 4 | 1 |
| Men <65 | 90 975 | 88 824 | 2151 | 1842 | 139 | 67 | 3 | 10 |
| Men ≥65 | 55 183 | 53 690 | 1493 | 1271 | 107 | 74 | 1 | 4 |
| All | 309 450 | 301 637 | 7813 | 6776 | 450 | 239 | 10 | 19 |

FIT= Fecal Immunochemical Test. SD CRC= screening detected CRC. IC= interval cancer. FIT IC= IC after negative FIT. Colonoscopy IC= IC after negative screening colonoscopy. IC rate= number of IC per 10 000 FIT negatives or FIT positives with negative or no colonoscopy. Test sensitivity = SD CRC/(SD CRC+FIT IC).

Supplementary Table 3. Estimations of FIT IC, IC rate and test sensitivity with cut-offs 80µg/g in both genders in different age and gender subgroups in the Stockholm-Gotland screening program for two screening rounds 2015-2017 and 2017-2019.

| Age at invitation and gender | FIT IC  2015-2017 | IC rate  2015-2017  * | Test sensitivity  2015-2017  ** | FIT IC  2017-2019 | IC rate  2017-2019  (95% CI)* | Test sensitivity  2017-2019  (95% CI)** | FIT IC  2015-2019 | IC rate  2015-2019  (95% CI)* | Test sensitivity  2015-2019  (95% CI)** |
| --- | --- | --- | --- | --- | --- | --- | --- | --- | --- |
| Women <65 | 37 | 8.2 (5.6-10.8) | 0.53 (0.42-0.64) | 38 | 8.0 (5.6-10.4) | 0.49 (0.38-0.61) | 75 | 8.1 (6.3-9.8) | 0.51 (0.43-0.59) |
| Women ≥65 | 35 | 11.4 (7.7-15.1) | 0.59 (0.49-0.70) | 40 | 12.7 (8.8-16.5) | 0.39 (0.28-0.51) | 75 | 12.0 (9.3-14.7) | 0.51 (0.43-0.59) |
| Men <65 | 32 | 8.6 (5.8-11.3) | 0.69 (0.60-0.78) | 35 | 9.0 (6.4-11.7) | 0.66 (0.56-0.75) | 67 | 8.8 (6.9-10.7) | 0.67 (0.61-0.74) |
| Men ≥65 | 43 | 16.2 (12.3-20.0) | 0.59 (0.50-0.69) | 31 | 12.6 (8.5-16.8) | 0.59 (0.48-0.70) | 74 | 14.3 (11.2-17.5) | 0.59 (0.52-0.66) |

FIT= Fecal Immunochemical Test. IC= interval cancer. FIT IC= IC after negative FIT. Colonoscopy IC= IC after negative screening colonoscopy. IC rate= number of IC (FIT IC, colonoscopy IC or IC in those non-compliant to colonoscopy) per 10 000 FIT negatives or FIT positives with negative or no colonoscopy. SD CRC= screening detected CRC. Test sensitivity = SD CRC/(SD CRC+FIT IC).

*) p-value= 0.265 ; 0.759 and 0.294 for difference in IC rate between men and women 2015-2017 ; 2017-2019 and 2015-2019 respectively.

**) p-value= 0.146 ; 0.0020 and 0.0011 for difference in test sensitivity between men and women 2015-2017 ; 2017-2019 and 2015-2019 respectively.

Supplementary Table 4. CRC characteristics in men and women invited to screening 2015-2019 by different modes of CRC detection.

| CRC characteristics | SD CRC  N (%) | FIT IC  N (%) | Colonoscopy IC  N (%) | IC non-compliant to colonoscopy  N (%) | Non-participants  N (%) |
| --- | --- | --- | --- | --- | --- |
| Total Women | 204 (48) | 98 (23) | 6 (1.4) | 5 (1.2) | 115 (27) |
| Men | 246 (40) | 141 (23) | 4 (0.7) | 14 (2.3) | 207 (34) |
|  |  |  |  |  |  |
| Stage I&II* Women | 114 (27) | 38 (8.9) | 4 (0.9) | 3 (0.7) | 34 (7.9) |
| Men | 146 (24) | 50 (8.2) | 3 (0.5) | 8 (1.3) | 73 (12) |
| Stage III&IV Women | 76 (18) | 46 (11) | 1 (0.2) | 2 (0.5) | 60 (14) |
| Men | 82 (13) | 66 (11) | 1 (0.2) | 6 (1.0) | 99 (16) |
| No stage Women | 14 (3.3) | 14 (3.3) | 1 (0.2) | 0 (0) | 21 (4.9) |
| Men | 18 (2.9) | 25 (4.1) | 0 (0) | 0 (0) | 35 (5.7) |
|  |  |  |  |  |  |
| pT1 Women | 39 (9.1) | 14 (3.3) | 3 (0.7) | 1 (0.2) | 3 (0.7) |
| Men | 61 (10) | 17 (2.8) | 2 (0.3) | 2 (0.3) | 13 (2.1) |
| pT2 Women | 48 (11) | 8 (1.9) | 1 (0.2) | 0 (0) | 8 (1.9) |
| Men | 45 (7.4) | 15 (2.5) | 0 (0) | 2 (0.3) | 22 (3.6) |
| pT3 Women | 71 (17) | 25 (5.8) | 1 (0.2) | 2 (0.5) | 34 (7.9) |
| Men | 83 (14) | 46 (7.5) | 2 (0.3) | 4 (0.7) | 62 (10) |
| pT4 Women | 18 (4.2) | 24 (5.6) | 1 (0.2) | 2 (0.5) | 34 (7.9) |
| Men | 13 (2.1) | 25 (4.1) | 0 (0) | 4 (0.7) | 49 (8.0) |
| No pT stage, TX, T0 Women | 28 (6.5) | 27 (6.3) | 0 (0) | 0 (0) | 36 (8.4) |
| Men | 44 (7.2) | 38 (6.2) | 0 (0) | 2 (0.3) | 61 (10) |
|  |  |  |  |  |  |
| Proximal** Women | 67 (16) | 56 (13) | 3 (0.7) | 3 (0.7) | 57 (13) |
| Men | 52 (8.5) | 60 (9.8) | 0 (0) | 4 (0.7) | 59 (9.6) |
| Distal Women | 135 (32) | 42 (9.8) | 3 (0.7) | 2 (0.5) | 58 (14) |
| Men | 193 (32) | 80 (13) | 4 (0.7) | 10 (1.6) | 148 (24) |
| No localization Women | 2 (0.5) | 0 (0) | 0 (0) | 0 (0) | 0 (0) |
| Men | 1 (0.2) | 1 (0.2) | 0 (0) | 0 (0) | 0 (0) |

CRC= colorectal cancer. Stage I&II = no regional lymph node metastasis, stage III&IV= regional lymph node or distant metastases. pT= histopathological T stage. Proximal= caecum to splenic flexure. Distal= descending colon to rectum. Percentages refer to proportion of all men and women with CRC respectively.

*) Stage I&II in participants vs non-participants, p-value =0.000009 (no stage, n=128, excluded).

**) Proximal localization in women as compared to men, p-value =0.0000009 (no localization, n=4, excluded).

Supplementary Table 5. FIT levels in men and women with SD CRCs and FIT ≥80µg/g.

| CRC characteristics | Women,  N | Men,  N | Women  FIT median,  (IQ range) | Men  FIT median,  (IQ range) | p-value  * |
| --- | --- | --- | --- | --- | --- |
| Total | 156 | 246 | 501.5 (159.8-1377.8) | 408.5 (167.2-1105.8) | 0.590 |
|  |  |  |  |  |  |
| Stage I&II | 87 | 146 | 394 (143-958.5) | 323.5 (158.2-850.5) | 0.976 |
| Stage III&IV | 63 | 82 | 790 (200.5-1659.5) | 637.5 (199.2-1848.8) | 0.929 |
| No stage | 6 | 18 | - | - | - |
|  |  |  |  |  |  |
| pT1 | 26 | 61 | 266.5 (132.8-963.2) | 193 (144-457) | 0.643 |
| pT2 | 31 | 45 | 254 (141-640) | 479 (176-889) | 0.254 |
| pT3 | 61 | 83 | 790 (201-1977) | 676 (242.5-1641.5) | 0.731 |
| pT4 | 17 | 13 | 817 (264-1765) | 673 (267-1457) | 0.867 |
| No pT stage, TX or T0 | 21 | 44 | - | - | - |
|  |  |  |  |  |  |
| No localization | 1 | 1 | - | - | - |
| Proximal | 49 | 52 | 283 (116-902) | 503 (146-1363) | 0.268 |
| Distal | 106 | 193 | 556 (193-1543) | 396 (169-1047) | 0.118 |

FIT= Fecal Immunochemical Test. CRC= colorectal cancer. SD CRC= screening detected CRC. Stage I&II= no regional lymph node metastasis, stage III&IV= regional lymph node or distant metastases. pT= histopathological T stage. Proximal= caecum to splenic flexure. Distal= descending colon to rectum. IQ range= interquartile range.

*) Wilcoxon rank sum test of FIT level in men *vs.* women.
